# Detailed Analysis of Back Pain: A Cross‐Sectional Study of Epidemiology and Previous Therapies in a German Population

**DOI:** 10.1111/papr.70072

**Published:** 2025-08-19

**Authors:** Bernhard U. Hoehl, Nima Taheri, Luis Alexander Becker, Lukas Mödl, Lukas Schönnagel, Christoph Stein, Matthias Pumberger, Hendrik Schmidt

**Affiliations:** ^1^ Center for Musculoskeletal Surgery Charité—Universitätsmedizin Berlin Berlin Germany; ^2^ Julius Wolff Institute Berlin Institute of Health at Charité—Universiätsmedizin Berlin Berlin Germany; ^3^ Institute of Biometry and Clinical Epidemiology Charité—Universitätsmedizin Berlin Berlin Germany; ^4^ Department of Experimental Anaesthesiology Charité—Universitätsmedizin Berlin Berlin Germany

**Keywords:** chronic, intermittent, LBP, low back pain

## Abstract

**Background:**

Diagnosing cLBP is complex due to its heterogeneity, lack of definitive biomarkers, and the subjective nature of pain. This study aims to analyze cLBP patients comprehensively, characterizing demographic and clinical profiles of patients and evaluating the types and effectiveness of previous treatments.

**Methods:**

From January 2022 to April 2024, we recruited 1262 participants aged between 18 and 72 years from the general population by advertisements and word‐of‐mouth propaganda. Collected data included a detailed medical history, standardized questionnaires (e.g., Von Korff) and a clinical examination.

**Results:**

Our population included 471 (38%) with chronic back pain, 335 (27%) without back pain, and an additional group in between of 327 (26%) with intermittent back pain. The majority of participants experienced multifocal back pain, which was further subdivided based on the predominant pain localization. In patients with localized cLBP (median: 5 years, IQR: 1–10 years), the pain persisted for a shorter time than in the group with predominant back pain other than the lower back (median: 10 years, IQR: 5–15 years). The majority of participants did either use no pain medication at all (cLBP: 32%; iLBP: 37%; no‐BP(2): 55%) or on‐demand medication (cLBP: 56%; iLBP: 56%; no‐BP(2): 39%).

**Conclusion:**

Our data show considerable heterogeneity underlying the widespread diagnosis “chronic back pain.” Quantitative differentiation is difficult due to the low pain intensity on the day of the examination, and adequate treatment recommendations are challenging. To better understand chronic back pain, there is a strong need for a subclassification.

**Trial Registration:**

German Clinical Trial Register: DRKS‐ID: DRKS00027907

## 1 Introduction

Chronic low back pain (cLBP) is a pervasive and often debilitating condition that affects millions of people worldwide and can significantly impact an individual's quality of life [1, 2, 3]. It is defined as pain that persists for 3 months or longer, even after an initial injury or other somatic cause of acute low back pain has been treated [4]. However, this definition is not very precise. For example, it is not clear whether it requires continuous or intermittent presence of pain, or how long the intervals between pain episodes are. The prevalence of cLBP varies widely across different populations, settings, and definitions [5]. Studies indicate that approximately 20% of people who experience acute low back pain will develop chronic symptoms [6]. The global prevalence of cLBP in adults is estimated to be around 18%, with variations based on age, sex, and geographic location [1]. cLBP significantly affects daily activities, leading to decreased mobility, sleep disturbances, and reduced ability to work [7, 8, 9]. It is further associated with psycho‐social issues such as sick leave, seclusion, depression, and anxiety [10].

The diagnosis of cLBP is fraught with challenges due to its complex and multifactorial nature. Currently, there are no definitive biomarkers for cLBP. Diagnostic imaging (e.g., MRI, CT scans) and other tests often show abnormalities that may not correlate with pain levels or may even be present in asymptomatic individuals [1, 11, 12, 13]. This lack of correlation can result in misdiagnosis or unnecessary treatments. Furthermore, pain is a subjective experience, varying significantly among individuals in terms of intensity, duration, and response to stimuli. This subjectivity makes it difficult to quantify and assess pain accurately, leading to potential underestimation or overestimation of the severity of cLBP.

Addressing these challenges requires a more detailed analysis of cLBP patients. Relevant factors include, but are not limited to, anatomical and physiological abnormalities, lifestyle, social and occupational influences, psychological stressors, and genetic predispositions. Furthermore, patient heterogeneity in terms of pain presentation, response to treatment, and overall prognosis necessitates a personalized approach to cLBP management [14].

The present study aims to conduct a comprehensive and detailed analysis of patients suffering from cLBP to better understand the multifactorial nature of the condition and to identify key factors that may contribute to its persistence. The specific objectives of the study are as follows:

1. To characterize the demographic and clinical profile of cLBP patients.
2. To evaluate treatment histories and outcomes.

## 2 Materials and Methods

### 2.1 Study Design

This prospective observational utilizes data from the ongoing “Berliner Rückenstudie” (“Berlin Back Study”; duration: 01/01/2022 to 31/12/2025), registered with the German Clinical Trial Register (DRKS‐ID: DRKS00027907). Recruitment methods include local advertisements at Charité‐University Hospital Berlin (flyers, notice boards, internet, social media), outreach to the general public (newspapers, magazines, podcasts, TV), collaboration with local companies, administrative offices, and word‐of‐mouth. The study protocol adheres to the ethical principles outlined in the Helsinki Declaration [15] and has been approved by the Ethics Committee of Charité‐Universitätsmedizin Berlin (registry numbers: EA1/058/21). All participants provided written informed consent. This study follows the Strengthening the Reporting of Observational Studies in Epidemiology (STROBE) guideline for reporting [16]. Data collection commenced on January 1, 2022, and the cut‐off for inclusion was April 24, 2024. Data collection took place at a specified research facility within the university hospital.

### 2.2 Study Participants—In‐ and Exclusion Criteria

Study participants were required to meet the following inclusion criteria: age 18–72 years and provide written informed consent to participate in the study; be either asymptomatic (no history of back, pelvis, or hip pain, and no spinal surgery) or symptomatic with cLBP with overall pain duration of at least 3 months.

Exclusion criteria included: professional, competitive, and top athletes; acute infections; substance abuse; pregnancy; body mass index (BMI) greater than 28 kg/m^2^ to ensure accurate surface measurement of the back [17], which is analyzed in another analysis; neurologic impairments (e.g., spinal cord injury, radicular symptoms, sensory deficits, paralysis); irritated, inflamed, or infected tissues in the back; spinal fractures; osteoporosis; tumors and bone metastases; previous spinal surgery; specific drug therapy (e.g., antiepileptics, long‐acting antihistamines, systemic glucocorticoids, or immunosuppressive drugs); rheumatic diseases; active systemic diseases (e.g., tuberculosis, collagenosis, multiple sclerosis, autoimmune diseases, acquired immune deficiency syndrome); internal diseases posing potential risks during measurements (e.g., coronary heart diseases, heart failure, malignant hypertension, chronic obstructive pulmonary disease); and malpositions or anomalies of the lower extremities (e.g., knee or hip arthroplasty, arthrodesis).

### 2.3 Quantitative Variables and Data Collection

A study coordinator provided concise instructions regarding the study protocol to the participants and escorted them through the following stages: (1) completion of 10 back pain‐related questionnaires, (2) medical history and physical examination by an orthopedic resident with multiple years of experience, and (3) assessment of back morphology and motion utilizing the Idiag M360 (MediMouse, Idiag AG, Fehraltorf, Switzerland). These examinations were conducted on the same day, averaging 90 min in total duration.

Demographic data as age, sex, body weight and height were recorded. Detailed information regarding the pain, including chronicity, its primary (=predominant area) and (if applicable) secondary (=less dominant) localization, pain duration and previous diagnoses and operations were obtained.

Pain intensity was assessed using the Numerical Rating Scale (NRS; 0–10) for current pain intensity at the time of examination, strongest pain in the past 3 months, and average pain of the past 3 months. The strongest and average pain experienced over the past 3 months was recorded based on the participants' recollections [18]. The characteristic pain intensity (CPI) was calculated using the mean NRS scores of current pain, average pain, and strongest pain; the chronic pain grade consisting of CPI and disability was assessed [18].

Common treatment histories were polled in categories including physiotherapy, transcutaneous electrical nerve stimulation (TENS), acupuncture, psychotherapy, infiltration, inpatient rehabilitation, and total number of different treatment types. The perceived therapy effect was documented on a three‐point Likert scale (yes, successful; partly successful; not successful).

Furthermore, the level of current pain medication, type of on‐demand pain medication, and its perceived effectiveness on a three‐point Likert scale (yes, successful; partly successful; not successful) were obtained.

### 2.4 Statistical Analysis

Descriptive analysis was performed by boxplots, violin plots, and bar plots. To compare statistical differences, the non‐parametric Kruskal–Wallis test was performed. The commonly used interpretation values for statistical effect size (Kruskal–Wallis *η* ^2^) in published literature are: 0.01 to < 0.06 (small effect), 0.06 to < 0.14 (moderate effect), and ≥ 0.14 (large effect) [19, 20]. The level of statistical significance was set at *p* = 0.05. The clinical effect size is discussed in the corresponding section. All statistical analyses were conducted using R software [21] and RStudio software Version 2023.12.1.402 [22].

## 3 Results

### 3.1 Study Population

**FIGURE 1:**
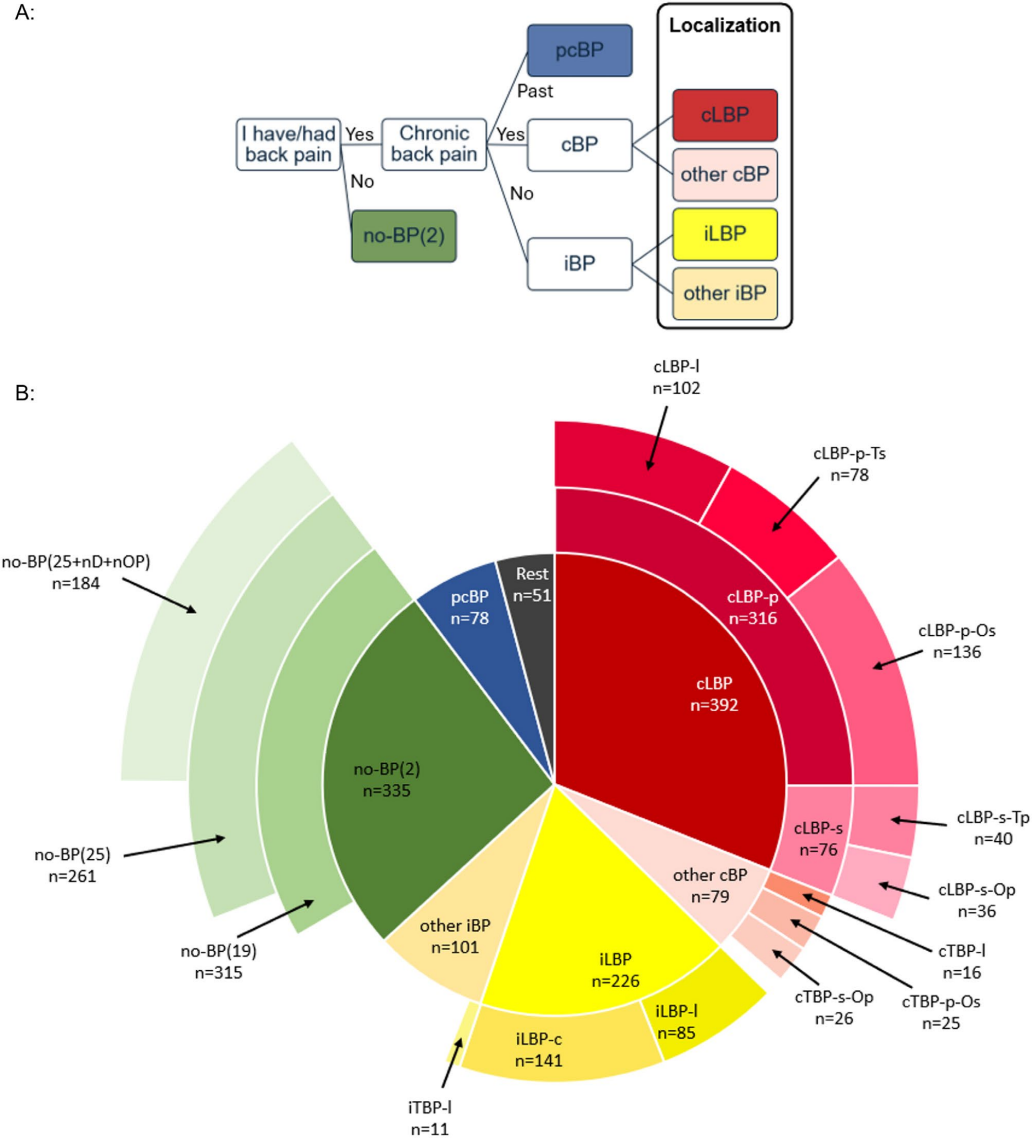
Flowchart of subclassification (A) and assignment of the 1262 participants to the subgroups (B). The inner circle illustrates the major groups, which are subdivided according to pain localization. The group without back pain (no‐BP) is further divided based on the number of considered items. …(*x*), number of considered items; ‐c, concomitant; cBP, chronic back pain; iBP, intermittent back pain; ‐l, localized; LBP, low back pain; nD, no diagnosis (spine/hip); no‐BP, no back pain; nOP, no operations (spine/leg); ‐Op, other primary (not T/L); ‐Os, other secondary (not T/L); ‐p, primary; pcBP, past chronic back pain; Rest, all others; ‐s, secondary; TBP, thoracic back pain; ‐Tp, thoracic primary; ‐Ts, thoracic secondary.

From January 2022 to April 2024, 1262 (male (m) = 545, female (f) = 716, divers “they/them” (d) = 1) participants were included. Up to 49% of participants reported experiencing chronic back pain. However, detailed history and physical examinations revealed a much broader range of complaints (Figure 1A,B). Accordingly, participants were divided into seven major groups (Figure 1B, inner circle): cLBP (*n* = 392, m = 172, f = 220), other chronic back pain (cBP) without involvement of the lower back (*n* = 79, m = 23, f = 55; d = 1), intermittent LBP (iLBP) with pain episodes shorter than 3 months (*n* = 226, m = 104, f = 122), other intermittent BP (iBP) without involvement of the lower back (*n* = 101, m = 52, f = 49). The no‐BP(2) group (*n* = 335, m = 140, f = 195) reported no back pain in response to the two initial questions. Seventy‐eight participants (m = 41, f = 37) with a history of past chronic back pain (pcBP) experienced at least one period of cBP characterized by daily pain lasting ≥ 3 months in the past. Fifty‐one participants (m = 13, f = 38) with pain in other areas or inconsistent responses were categorized as “Rest.” Focusing on LBP, the three associated major groups were divided into subgroups: cLBP was subdivided based on primary (cLBP‐p) and secondary (cLBP‐s) pain localization. The localized cLBP group (cLBP‐l: *n* = 102, m = 59, f = 43) experienced chronic pain solely in the lower back. The cLBP‐p‐Ts group (*n* = 78, m = 31, f = 47) reported primary LBP with secondary thoracic back pain. The iLBP group was subdivided into: pain localized only in the lower back (iLBP‐l: *n* = 85, m = 49, f = 36) and concomitant pain (iLBP‐c: *n* = 141, m = 55, f = 86).

The no‐BP(2) group was further divided into the following subgroups based on responses to 17 additional questions related to back pain (2 + 17 = 19): the no‐BP(19) group (*n* = 315, m = 132, f = 183) denied experiencing any back pain, the no‐BP(25) group (*n* = 261, m = 110, f = 151) reported no pain in 6 additional pain‐specific clinical tests (2 + 17 + 6 = 25), and the no‐BP(25 + nD + nOP) (*n* = 184, m = 79, f = 105) also had no documented diagnosis of spinal or lower extremity conditions and no history of spinal or leg surgery.

### 3.2 Demographic Data

**FIGURE 2:**
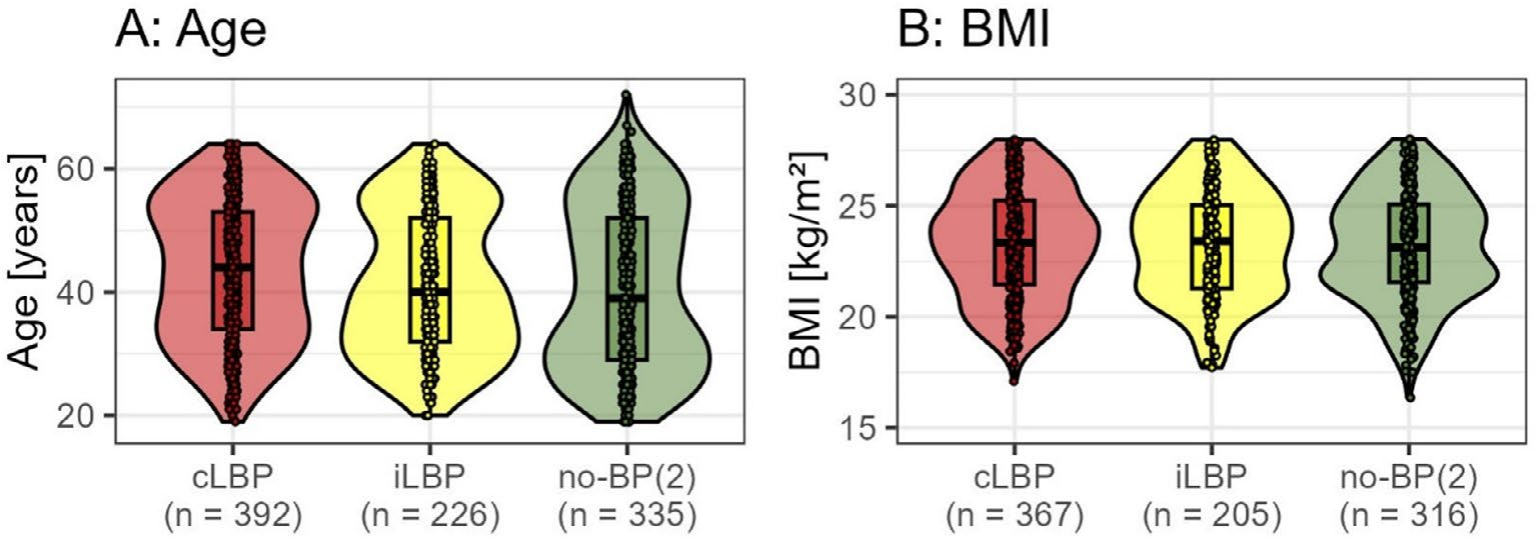
Comparison of age (A) and body mass index (BMI) (B) of the major groups with chronic low back pain (cLBP), intermittent low back pain (iLBP) and without back pain (no‐BP(2)).

Between the major groups (inner circle, Figure 1B), there was a statistically significant but small difference in age with a trend towards an older age in the cLBP group (*p* = 0.001, Kruskal–Wallis *η* ^2^ = 0.014) and no significant difference in sex (*p* = 0.609) or BMI (*p* = 0.805) (Figure 2A,B). Within the subgroups, there were significantly more female patients in iLBP‐c (61%) than in iLBP‐l (42%) (*η* ^2^ = 0.033; *p* = 0.007) and fewer female subjects in cLBP‐l (42%) than in the other minor groups of cLBP (61%) (*η* ^2^ = 0.030; *p* = 0.021). There were no other significant differences in the minor groups regarding age (cLBP: *η* ^2^ = 0.018; *p* = 0.129; iLBP: *η* ^2^ = 0.003; *p* = 0.384) or BMI (cLBP: *η* ^2^ = 0.013; *p* = 0.321; iLBP: *η* ^2^ = 0.001; *p* = 0.736).

### 3.3 Pain Intensity

**FIGURE 3:**
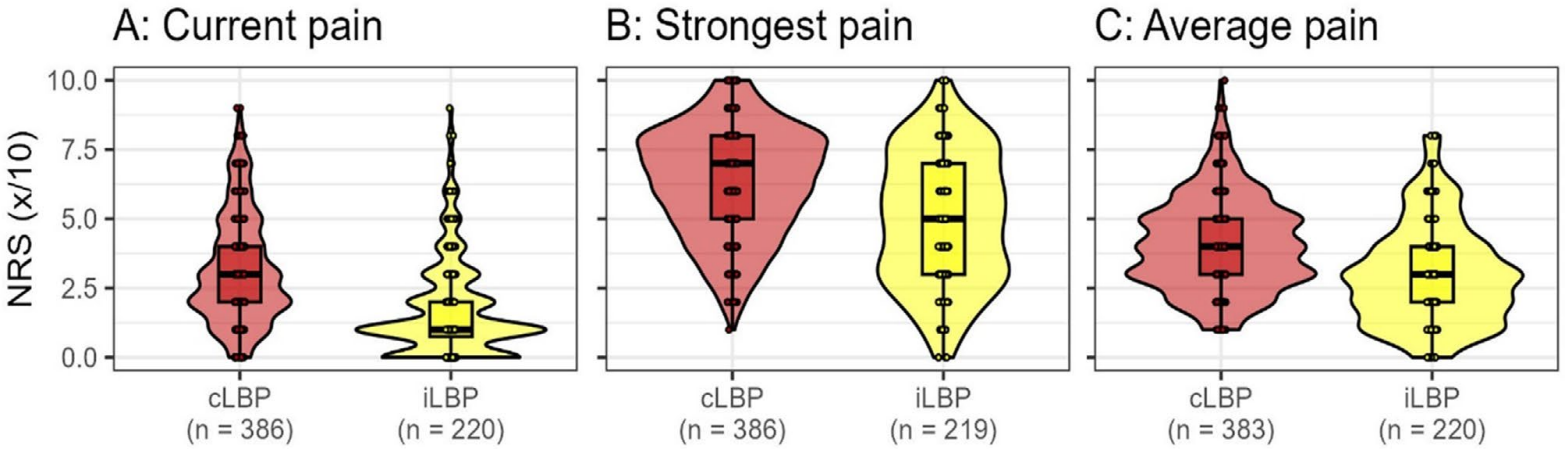
Comparison of the current pain intensity during examination (A), highest pain experienced in the past 3 months (B), and the average pain level of the past 3 months (C) of the groups with chronic low back pain (cLBP) and intermittent low back pain (iLBP) on the numerous rating scale (NRS 0–10). Participants with no back pain (*n* = 335) are not shown.

Pain levels were significantly higher in the cLBP than in the iLBP group across all measured domains: current pain intensity at the time of examination (cLBP: median: 3, IQR: 2–4; iLBP: median: 1, IQR: 0.75–2; *η* ^2^ = 0.151, *p* < 0.001), strongest pain (cLBP: median: 7, IQR: 5–8; iLBP: median: 5, IQR: 3–7; *η* ^2^ = 0.077, *p* < 0.001), and average pain (cLBP: median: 4, IQR: 3–5; iLBP: median: 3, IQR: 2–4; *η* ^2^ = 0.092; *p* < 0.001) (Figure 3A–C).

The current pain intensity during examination was median 3 (IQR 2–5) points lower than the highest pain experienced in the past 3 months and 1 (IQR 0–2) point lower than the average pain level during that period. The only significant difference observed between the groups was the disparity between current pain and average pain in the main groups (cLBP: median = 1, IQR = 0–2; iLBP: median = 1, IQR = 0–2; *η* ^2^ = 0.008, *p* = 0.029).

### 3.4 Chronic Pain Grade and Characteristic Pain Intensity

**FIGURE 4:**
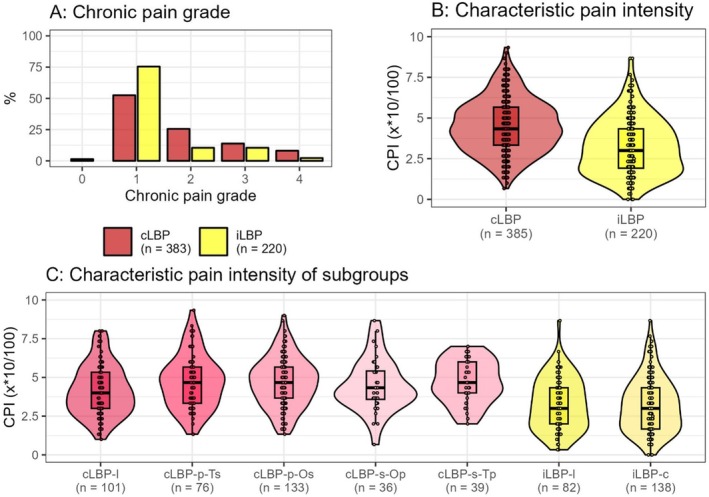
Chronic pain grade (A), characteristic pain intensity (CPI) (B) of the main groups (inner circle, Figure 1B) and CPI of the subgroups (C) according to Von Korff et al. [18]. cLBP, chronic low back pain; cLBP‐p‐Os, primary cLBP, secondary other pain (not thoracic or low back); cLBP‐p‐Ts, primary cLBP, secondary pain in the thoracic back; cLBP‐s‐Op, secondary cLBP, primary other pain (not thoracic or low back); cLBP‐s‐Tp, secondary cLBP, primary thoracic back; iLBP‐c, concomitant iLBP; iLBP‐l, localized iLBP.

The chronic pain grade (*η* ^2^ = 0.055, *p* < 0.001) and characteristic pain intensity (CPI) [18] exhibited a significant difference between the primary groups cLBP and iLBP (inner circle, Figure 1B) [18]. Specifically, cLBP showed a median CPI of 43 (IQR 33–57), whereas iLBP had a median CPI of 30 (IQR: 13–43) (*η* ^2^ = 0.126, *p* < 0.001). No significant differences were found within the subgroups of cLBP (*η* ^2^ = 0.023, *p* = 0.068) or iLBP (*η* ^2^ = 0.001, *p* = 0.625). The most pronounced difference was observed between participants with chronic secondary LBP and primary thoracic pain (cLBP‐s‐Tp: median = 47, IQR 40–60) and those with concomitant iLBP (iLBP‐c: median = 30, IQR 17–43) (*η* ^2^ = 0.139, *p* < 0.001) (Figure 4A–C).

### 3.5 Pain Duration

**FIGURE 5:**
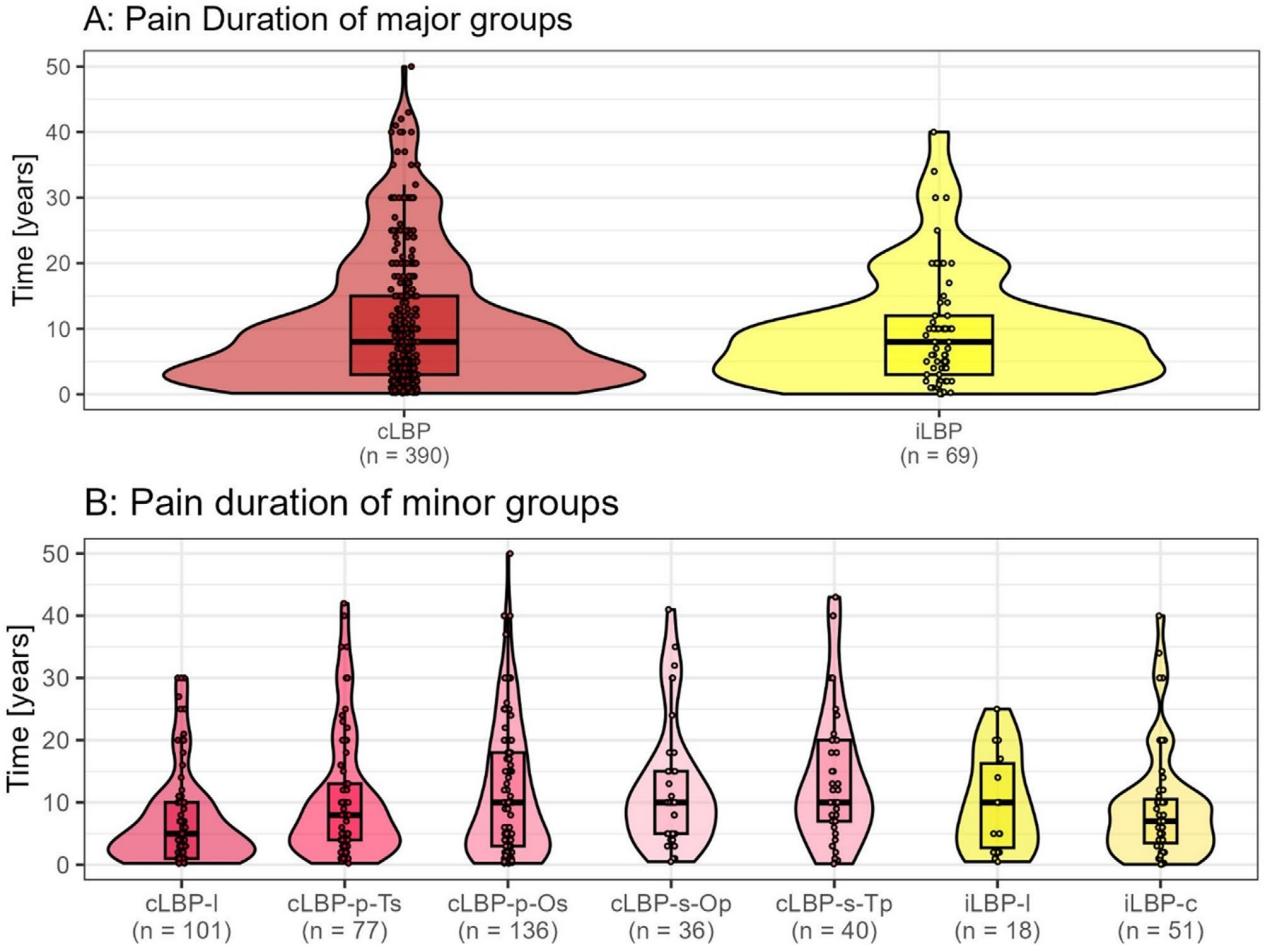
Pain duration of the major groups (A) and minor groups (B) in years. cLBP, chronic low back pain; cLBP‐p‐Os, primary cLBP, secondary other pain (not thoracic or low back); cLBP‐p‐Ts, primary cLBP, secondary pain in the thoracic back; cLBP‐s‐Op, secondary cLBP, primary other pain (not thoracic or low back); cLBP‐s‐Tp, secondary cLBP, primary thoracic back; iLBP‐c, concomitant iLBP; iLBP‐l, localized iLBP.

There was no significant difference observed in the pain duration in years (given as a retrospective assessment by the subjects) between the major groups (cLBP: median = 8, IQR 3–15; iLBP: median = 7, IQR 3–11; *η* ^2^ = 0.000, *p* = 0.754). Looking at the minor groups, cLBP‐l showed a significantly shorter pain duration (median = 5, IQR 1–10) than, for example, cLBP‐p‐Os (median = 10, IQR 3–18) (*η* ^2^ = 0.056, *p* < 0.001) (Figure 5A,B).

### 3.6 Previous Therapies

**FIGURE 6:**
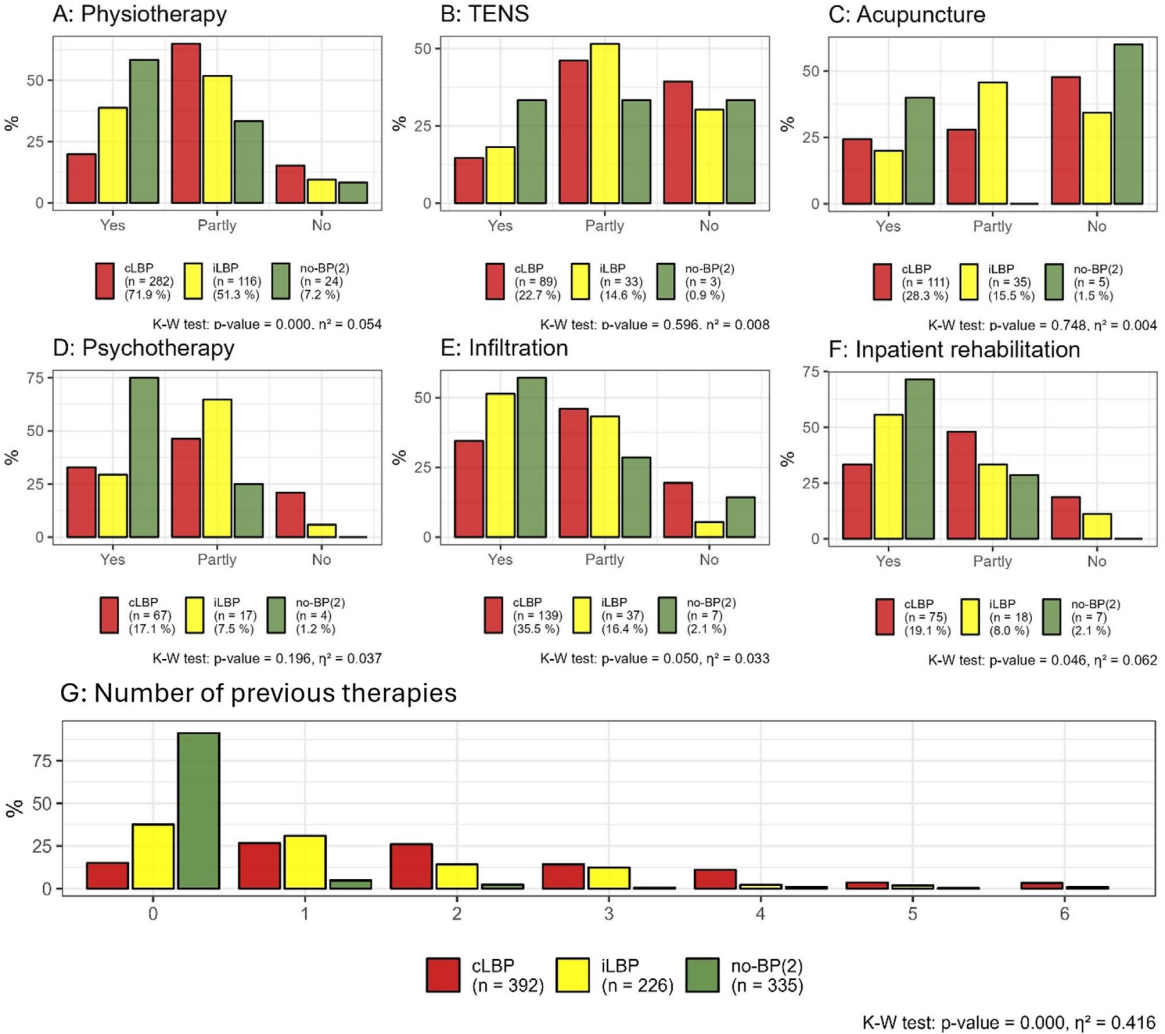
The types of applied therapies and their effects (3‐point‐Likert scale: Yes, partly, no) are displayed for the groups with chronic low back pain (cLBP), intermittent low back pain (iLBP), and no back pain (no‐BP(2)) (A–F). For each group, the number (*n*) of participants who applied the therapy and the percentage share of each group are indicated. The majority of cLBP applied more therapies than iLBP (G). K–W test, Kruskal–Wallis test.

The predominant therapy utilized was physiotherapy (cLBP: 72%; iLBP: 51%). Infiltrations demonstrated the most significant effect (> 50%) within the iLBP group. cLBP (median = 2, IQR: 1–3) underwent significantly more previous therapies (*η* ^2^ = 0.416, *p* < 0.001) than iLBP (median = 1, IQR: 0–2) or no‐BP(2) (median = 0, IQR: 0–0) (Figure 6A–G).

### 3.7 Current Medication

**FIGURE 7:**
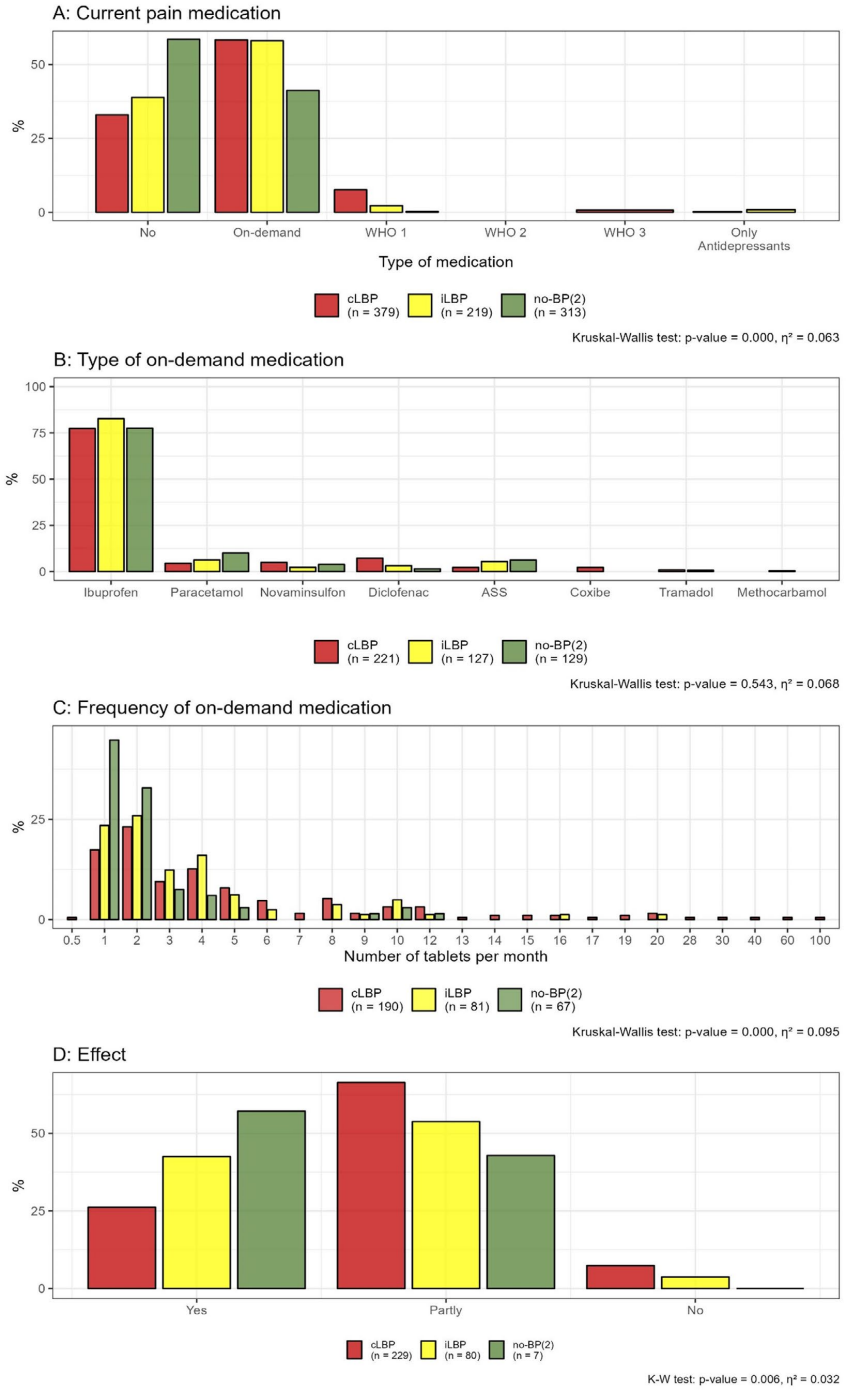
Pain medication (A), type of on‐demand medication (B), number of tablets taken per month (C) and the perceived effect of the total medication (D) for chronic low back pain (cLBP), intermittent low back pain (iLBP) and no back pain (no‐BP(2)). Kruskal–Wallis test (K–W test) was used to analyze significant differences (*p*‐value) and effect sizes (*η* ^2^).

The majority of participants did not take pain medication on a daily basis. Among the groups, 32% of individuals with cLBP, 37% with iLBP, and 55% without back pain (no‐BP(2)) did not use any pain medication. On‐demand pain medication (PRN = pro re nata) was used by 56% of both the cLBP and iLBP groups, and by 39% of the no‐BP(2) group. Overall, Ibuprofen was the most frequently on‐demand medication at 79%, followed by paracetamol (7.3%), metamizole (4.5%), and diclofenac (4%). The majority of patients required an average of up to 5 tablets per month (Figure 7A–D).

## 4 Discussion

This study provides a comprehensive analysis of cLBP in a cohort of 1262 participants, offering new insights into the demographic, clinical, and treatment profiles of those affected by this debilitating condition. The study identified significant differences in pain intensity, chronic pain grade, and previous therapy utilization between participants with cLBP and those with iLBP. Notably, cLBP patients reported higher pain levels across all measures, with a more severe CPI and a greater number of previous therapies compared to iLBP patients. Despite these differences, no significant variations were found in pain duration between the major groups, although certain subgroups within cLBP showed distinct patterns.

Current pain intensity, although a widely used and easily accessible variable, may not fully represent overall perceived pain due to potential variations. To address these limitations, Von Korff et al. introduced the CPI, which includes average pain and the strongest pain over the past 3 months, in addition to current pain. Despite the limitations of reporting recalled pain intensities over 90 days compared to daily assessments, the CPI has been shown to be more consistent than current pain intensity alone [18, 23]. Clinically significant differences in pain are typically considered to be at least 2 points on a 10‐point scale, or about 30% [24, 25], with changes of 4 points or 50% considered as “very much improved/worsened” [26]. The significantly higher pain intensity observed in the cLBP group across all measures, including current, strongest, and average pain, aligns with existing literature that describes cLBP as more persistent and severe than its intermittent counterpart [27, 28]. The finding that cLBP patients had a higher median CPI further supports the notion that chronic pain is not only more intense but also more disabling, affecting daily activities and quality of life to a greater extent than intermittent pain [28].

The chronic pain grade, which combines CPI with disability measures, revealed substantial differences between cLBP and iLBP groups, particularly in those with secondary thoracic pain. This suggests that the presence of secondary pain locations exacerbates the overall pain experience and disability in cLBP patients. The lack of significant differences within the subgroups of cLBP and iLBP for pain intensity and duration might indicate that once pain becomes chronic, other factors such as psychological and social influences play a more pivotal role in patient experience than the mere duration of symptoms [10].

The analysis of treatment histories revealed that cLBP patients underwent significantly more therapeutic interventions than those with iLBP or no back pain, which may reflect the chronic and refractory nature of their condition. The predominance of physiotherapy among treatments is consistent with current guidelines recommending physical therapy as a cornerstone of cLBP management [29]. However, the limited success of these interventions, as indicated by the relatively high number of previous therapies and continued high pain levels, underscores the need for more effective and tailored treatment strategies for cLBP [30, 31].

Interestingly, infiltration therapies showed significant effectiveness in the iLBP group, suggesting that certain interventions may be more beneficial in earlier or less chronic stages of LBP. This highlights the importance of early and appropriate intervention to prevent the progression to chronicity [32].

The study found that a substantial proportion of participants did not use regular pain medication, with on‐demand medication being more common. Ibuprofen was the most frequently used on‐demand medication, which is in line with its widespread use for musculoskeletal pain due to its anti‐inflammatory properties [33]. The relatively low reliance on regular medication may reflect concerns about long‐term use of analgesics or the inadequacy of existing pain management options for cLBP [29, 34].

The findings of this study have several implications for clinical practice. The high prevalence of cLBP and its association with greater pain intensity and disability emphasize the need for early identification and intervention to prevent the transition from acute to chronic pain. Additionally, the significant use of multiple therapies among cLBP patients suggests that a more personalized and multimodal approach may be necessary to manage this condition effectively. Clinicians should also consider the potential benefits of alternative therapies, such as infiltrations, particularly in cases of intermittent or less chronic pain.

While this study provides valuable insights into cLBP, it also has limitations that should be considered. The observational nature of the study limits the ability to establish causal relationships between the variables studied. Furthermore, the reliance on self‐reported data for pain intensity and treatment efficacy may introduce recall bias. Future research should aim to incorporate longitudinal designs to better understand the progression of cLBP and the long‐term effectiveness of various treatment modalities. The overall predominance of females in our study (males: 545, females: 716, divers: 1) might be attributed to the recruitment process. It included advertisements on social media, institutional websites, and public institutions, potentially reaching more females willing to participate in a multi‐hour trial. Although previous literature indicates a higher prevalence of back pain among females [1], our study of voluntary participants found no significant sex differences in our group distributions (*p* = 0.609). Our study included participants aged 18 to 72 years, aligning with other large‐scale epidemiological studies on cLBP. A statistically significant but clinically negligible age difference was observed between the major groups. Hoy et al. [35] noted that the highest prevalence of back pain occurs between 40 and 80 years of age [35]. Whereas previous studies showed that obesity significantly affects the mechanical dynamics of the lumbar spine [36, 37, 38], our study found no significant difference in BMI between groups (*p* = 0.805). This might be due to our exclusion criteria, which disqualified participants with a BMI greater than 28 kg/m^2^ to ensure reliable results from an electronic measurement device for spinal shape [17]. These data will be reported in a separate publication.

In conclusion, this study contributes to the growing body of evidence on cLBP, highlighting its complexity and the need for more effective, individualized treatment strategies. By addressing the multifactorial nature of cLBP, future interventions can be better tailored to meet the needs of this challenging patient population.

## Author Contributions

Acquisition: Bernhard U. Hoehl, Nima Taheri, Luis Alexander Becker, and Lukas Schönnagel. Analysis: Bernhard U. Hoehl, Lukas Mödl, and Hendrik Schmidt. Interpretation: Bernhard U. Hoehl, Nima Taheri, Luis Alexander Becker, Lukas Mödl, Lukas Schönnagel, Christoph Stein, Matthias Pumberger, and Hendrik Schmidt. All Authors contributed substantially.

## Ethics Statement

The study has been approved by the Ethics Committee of Charité‐Universitätsmedizin Berlin (registry numbers: EA1/058/21).

## Consent

All Participants were at least 18 years old and provided written informed consent.

## Conflicts of Interest

The authors declare no conflicts of interest.

## Supporting information


**Data S1:** papr70072‐sup‐0001‐Supinfo.doc.

## Data Availability

The data that support the findings of this study are available from the corresponding author upon reasonable request.

## References

[1] C. Maher , M. Underwood , and R. Buchbinder , “Non‐Specific Low Back Pain,” Lancet 389, no. 10070 (2017): 736–747, 10.1016/s0140-6736(16)30970-9. 27745712

[2] X. Pericot‐Mozo , R. Suñer‐Soler , G. Reig‐Garcia , et al., “Quality of Life in Patients With Chronic Low Back Pain and Differences by Sex: A Longitudinal Study,” Journal of Personalized Medicine 14, no. 5 (2024): 496, 10.3390/jpm14050496. 38793078 PMC11121820

[3] T. Vos , A. Abajobir , C. Abbafati , et al., “Global, Regional, and National Incidence, Prevalence, and Years Lived With Disability for 328 Diseases and Injuries for 195 Countries, 1990–2016: A Systematic Analysis for the Global Burden of Disease Study 2016,” Lancet 390, no. 10100 (2017): 1211–1259, 10.1016/s0140-6736(17)32154-2. 28919117 PMC5605509

[4] S. Perrot , M. Cohen , A. Barke , B. Korwisi , W. Rief , and R. D. Treede , “The IASP Classification of Chronic Pain for ICD‐11: Chronic Secondary Musculoskeletal Pain,” Pain 160, no. 1 (2019): 77–82, 10.1097/j.pain.0000000000001389. 30586074

[5] T. Jackson , S. Thomas , V. Stabile , M. Shotwell , X. Han , and K. McQueen , “A Systematic Review and Meta‐Analysis of the Global Burden of Chronic Pain Without Clear Etiology in Low‐ and Middle‐Income Countries: Trends in Heterogeneous Data and a Proposal for New Assessment Methods,” Anesthesia and Analgesia 123, no. 3 (2016): 739–748, 10.1213/ane.0000000000001389. 27537761

[6] R. D. Meucci , A. G. Fassa , and N. M. Faria , “Prevalence of Chronic Low Back Pain: Systematic Review,” Revista de Saúde Pública 49, no. 1 (2015): 73, 10.1590/s0034-8910.2015049005874. PMC4603263 26487293

[7] Z. Goossens , T. Bilterys , E. Van Looveren , et al., “The Role of Anxiety and Depression in Shaping the Sleep‐Pain Connection in Patients With Nonspecific Chronic Spinal Pain and Comorbid Insomnia: A Cross‐Sectional Analysis,” Journal of Clinical Medicine 13, no. 5 (2024): 1452, 10.3390/jcm13051452. 38592310 PMC10932262

[8] H. Miranda , E. Viikari‐Juntura , L. Punnett , and H. Riihimäki , “Occupational Loading, Health Behavior and Sleep Disturbance as Predictors of Low‐Back Pain,” Scandinavian Journal of Work, Environment & Health 34, no. 6 (2008): 411–419, 10.5271/sjweh.1290. 19137202

[9] J. S. Trinderup , A. Fisker , C. B. Juhl , and T. Petersen , “Fear Avoidance Beliefs as a Predictor for Long‐Term Sick Leave, Disability and Pain in Patients With Chronic Low Back Pain,” BMC Musculoskeletal Disorders 19, no. 1 (2018): 431, 10.1186/s12891-018-2351-9. 30509231 PMC6278039

[10] M. K. Nicholas , S. J. Linton , P. J. Watson , and C. J. Main , “Early Identification and Management of Psychological Risk Factors (‘Yellow Flags’) in Patients With Low Back Pain: A Reappraisal,” Physical Therapy 91, no. 5 (2011): 737–753, 10.2522/ptj.20100224. 21451099

[11] R. A. Deyo and J. N. Weinstein , “Low Back Pain,” New England Journal of Medicine 344, no. 5 (2001): 363–370, 10.1056/nejm200102013440508. 11172169

[12] E. T. Maas , J. N. Juch , R. W. Ostelo , et al., “Systematic Review of Patient History and Physical Examination to Diagnose Chronic Low Back Pain Originating From the Facet Joints,” European Journal of Pain 21, no. 3 (2017): 403–414, 10.1002/ejp.963. 27723170

[13] G. Pransky , R. Buchbinder , and J. Hayden , “Contemporary Low Back Pain Research—And Implications for Practice,” Best Practice & Research. Clinical Rheumatology 24, no. 2 (2010): 291–298, 10.1016/j.berh.2010.01.001. 20227649

[14] M. C. Mauck , A. F. Aylward , C. E. Barton , et al., “Evidence‐Based Interventions to Treat Chronic Low Back Pain: Treatment Selection for a Personalized Medicine Approach,” Pain Reports 7, no. 5 (2022): e1019, 10.1097/pr9.0000000000001019. 36203645 PMC9529058

[15] World Medical Association , “World Medical Association Declaration of Helsinki: Ethical Principles for Medical Research Involving Human Subjects,” Journal of the American Medical Association 310, no. 20 (2013): 2191–2194, 10.1001/jama.2013.281053. 24141714

[16] E. von Elm , D. G. Altman , M. Egger , S. J. Pocock , P. C. Gøtzsche , and J. P. Vandenbroucke , “The Strengthening the Reporting of Observational Studies in Epidemiology (STROBE) Statement: Guidelines for Reporting Observational Studies,” Lancet 370, no. 9596 (2007): 1453–1457, 10.1016/s0140-6736(07)61602-x. 18064739

[17] M. Guermazi , S. Ghroubi , M. Kassis , et al., “Validity and Reliability of Spinal Mouse to Assess Lumbar Flexion [Validité et reproductibilité du Spinal Mouse pour l'étude de la mobilité en flexion du rachis lombaire],” Annales de Réadaptation et de Médecine Physique 49, no. 4 (2006): 172–177, 10.1016/j.annrmp.2006.03.001. 16630669

[18] M. Von Korff , J. Ormel , F. J. Keefe , and S. F. Dworkin , “Grading the Severity of Chronic Pain,” Pain 50, no. 2 (1992): 133–149, 10.1016/0304-3959(92)90154-4. 1408309

[19] J. Cohen and J. W. Cohen , Statistical Power Analysis for the Behavioral Sciences, 2nd ed. (Erlbaum, 1988).

[20] M. Tomczak and E. Tomczak‐Łukaszewska , “The Need to Report Effect Size Estimates Revisited. An Overview of Some Recommended Measures of Effect Size,” Trends in Sport Sciences 21 (2014): 19–25.

[21] R Core Team , R: A Language and Environment for Statistical Computing (R Foundation for Statistical Computing, 2023), https://www.R‐project.org/.

[22] Posit Team , Rstudio: Integrated Development Environment for R (Posit Software, 2024), http://www.posit.co/.

[23] S. F. Dworkin , M. Von Korff , C. W. Whitney , L. Le Resche , B. G. Dicker , and W. Barlow , “Measurement of Characteristic Pain Intensity in Field Research,” Pain 41 (1990): S290, 10.1016/0304-3959(90)92696-N.

[24] M. F. Olsen , E. Bjerre , M. D. Hansen , B. Tendal , J. Hilden , and A. Hróbjartsson , “Minimum Clinically Important Differences in Chronic Pain Vary Considerably by Baseline Pain and Methodological Factors: Systematic Review of Empirical Studies,” Journal of Clinical Epidemiology 101 (2018): 87–106, 10.1016/j.jclinepi.2018.05.007. 29793007

[25] K. Peterson , J. Anderson , D. Bourne , K. Mackey , and M. Helfand , “Effectiveness of Models Used to Deliver Multimodal Care for Chronic Musculoskeletal Pain: A Rapid Evidence Review,” Journal of General Internal Medicine 33, no. S1 (2018): 71–81, 10.1007/s11606-018-4328-7. 29633140 PMC5902347

[26] R. H. Dworkin , D. C. Turk , K. W. Wyrwich , et al., “Interpreting the Clinical Importance of Treatment Outcomes in Chronic Pain Clinical Trials: IMMPACT Recommendations,” Journal of Pain 9, no. 2 (2008): 105–121, 10.1016/j.jpain.2007.09.005. 18055266

[27] L. d. C. Menezes Costa , C. G. Maher , M. J. Hancock , J. H. McAuley , R. D. Herbert , and L. O. Costa , “The Prognosis of Acute and Persistent Low‐Back Pain: A Meta‐Analysis,” Canadian Medical Association Journal 184, no. 11 (2012): E613–E624, 10.1503/cmaj.111271. 22586331 PMC3414626

[28] S. B. Wallwork , F. A. Braithwaite , M. O'Keeffe , et al., “The Clinical Course of Acute, Subacute and Persistent Low Back Pain: A Systematic Review and Meta‐Analysis,” Canadian Medical Association Journal 196, no. 2 (2024): E29–e46, 10.1503/cmaj.230542. 38253366 PMC10805138

[29] A. C. Traeger , R. Buchbinder , I. A. Harris , O. M. Clavisi , and C. G. Maher , “Avoid Routinely Prescribing Medicines for Non‐Specific Low Back Pain,” British Journal of Sports Medicine 53, no. 3 (2019): 196–199, 10.1136/bjsports-2017-098614. 29514824

[30] F. L. Bishop , A. L. Dima , J. Ngui , et al., “‘Lovely Pie in the Sky Plans’: A Qualitative Study of Clinicians' Perspectives on Guidelines for Managing Low Back Pain in Primary Care in England,” Spine 40, no. 23 (2015): 1842–1850, 10.1097/brs.0000000000001215. 26571064

[31] N. N. Knezevic , K. D. Candido , J. W. S. Vlaeyen , J. Van Zundert , and S. P. Cohen , “Low Back Pain,” Lancet 398, no. 10294 (2021): 78–92, 10.1016/s0140-6736(21)00733-9. 34115979

[32] N. E. Foster , J. R. Anema , D. Cherkin , et al., “Prevention and Treatment of Low Back Pain: Evidence, Challenges, and Promising Directions,” Lancet 391, no. 10137 (2018): 2368–2383, 10.1016/s0140-6736(18)30489-6. 29573872

[33] F. Migliorini , N. Maffulli , J. Eschweiler , et al., “The Pharmacological Management of Chronic Lower Back Pain,” Expert Opinion on Pharmacotherapy 22, no. 1 (2021): 109–119, 10.1080/14656566.2020.1817384. 32885995

[34] M. L. Ferreira , R. D. Herbert , P. H. Ferreira , et al., “The Smallest Worthwhile Effect of Nonsteroidal Anti‐Inflammatory Drugs and Physiotherapy for Chronic Low Back Pain: A Benefit‐Harm Trade‐Off Study,” Journal of Clinical Epidemiology 66, no. 12 (2013): 1397–1404, 10.1016/j.jclinepi.2013.02.018. 24021611

[35] D. Hoy , C. Bain , G. Williams , et al., “A Systematic Review of the Global Prevalence of Low Back Pain,” Arthritis and Rheumatism 64, no. 6 (2012): 2028–2037, 10.1002/art.34347. 22231424

[36] N. A. Abd Rahman , S. Li , S. Schmid , and S. Shaharudin , “Biomechanical Factors Associated With Non‐Specific Low Back Pain in Adults: A Systematic Review,” Physical Therapy in Sport 59 (2023): 60–72, 10.1016/j.ptsp.2022.11.011. 36516512

[37] J. A. Coppock , S. T. Danyluk , Z. A. Englander , C. E. Spritzer , A. P. Goode , and L. E. DeFrate , “Increasing BMI Increases Lumbar Intervertebral Disc Deformation Following a Treadmill Walking Stress Test,” Journal of Biomechanics 121 (2021): 110392, 10.1016/j.jbiomech.2021.110392. 33819699 PMC8128153

[38] R. Shiri , J. Karppinen , P. Leino‐Arjas , S. Solovieva , and E. Viikari‐Juntura , “The Association Between Obesity and Low Back Pain: A Meta‐Analysis,” American Journal of Epidemiology 171, no. 2 (2010): 135–154, 10.1093/aje/kwp356. 20007994

